# Internal initiation of reverse transcription in a Penelope-like retrotransposon

**DOI:** 10.1186/s13100-024-00322-z

**Published:** 2024-06-11

**Authors:** Chris J. Frangieh, Max E. Wilkinson, Daniel Strebinger, Jonathan Strecker, Michelle L. Walsh, Guilhem Faure, Irina A. Yushenova, Rhiannon K. Macrae, Irina R. Arkhipova, Feng Zhang

**Affiliations:** 1 Howard Hughes Medical Institute, Cambridge, MA 02139 USA; 2 Broad Institute of MIT and Harvard, Cambridge, MA 02142 USA; 3 McGovern Institute for Brain Research, Massachusetts Institute of Technology, Cambridge, MA 02139 USA; 4 Department of Brain and Cognitive Science, Massachusetts Institute of Technology, Cambridge, MA 02139 USA; 5 Department of Biological Engineering, Massachusetts Institute of Technology, Cambridge, MA 02139 USA; 6 Department of Electrical Engineering and Computer Science, Massachusetts Institute of Technology, Cambridge, MA 02139 USA; 7 Josephine Bay Paul Center for Comparative Molecular Biology and Evolution, Marine Biological Laboratory, Woods Hole, MA 02543 USA

**Keywords:** Reverse transcriptase, GIY-YIG endonuclease, Hammerhead ribozyme

## Abstract

Eukaryotic retroelements are generally divided into two classes: long terminal repeat (LTR) retrotransposons and non-LTR retrotransposons. A third class of eukaryotic retroelement, the Penelope-like elements (PLEs), has been well-characterized bioinformatically, but relatively little is known about the transposition mechanism of these elements. PLEs share some features with the R2 retrotransposon from *Bombyx mori*, which uses a target-primed reverse transcription (TPRT) mechanism, but their distinct phylogeny suggests PLEs may utilize a novel mechanism of mobilization. Using protein purified from *E. coli*, we report unique in vitro properties of a PLE from the green anole (*Anolis carolinensis*), revealing mechanistic aspects not shared by other retrotransposons. We found that reverse transcription is initiated at two adjacent sites within the transposon RNA that is not homologous to the cleaved DNA, a feature that is reflected in the genomic “tail” signature shared between and unique to PLEs. Our results for the first active PLE in vitro provide a starting point for understanding PLE mobilization and biology.

**Supplementary Information:**

The online version contains supplementary material available at 10.1186/s13100-024-00322-z.

## Main text

**Fig. 1:**
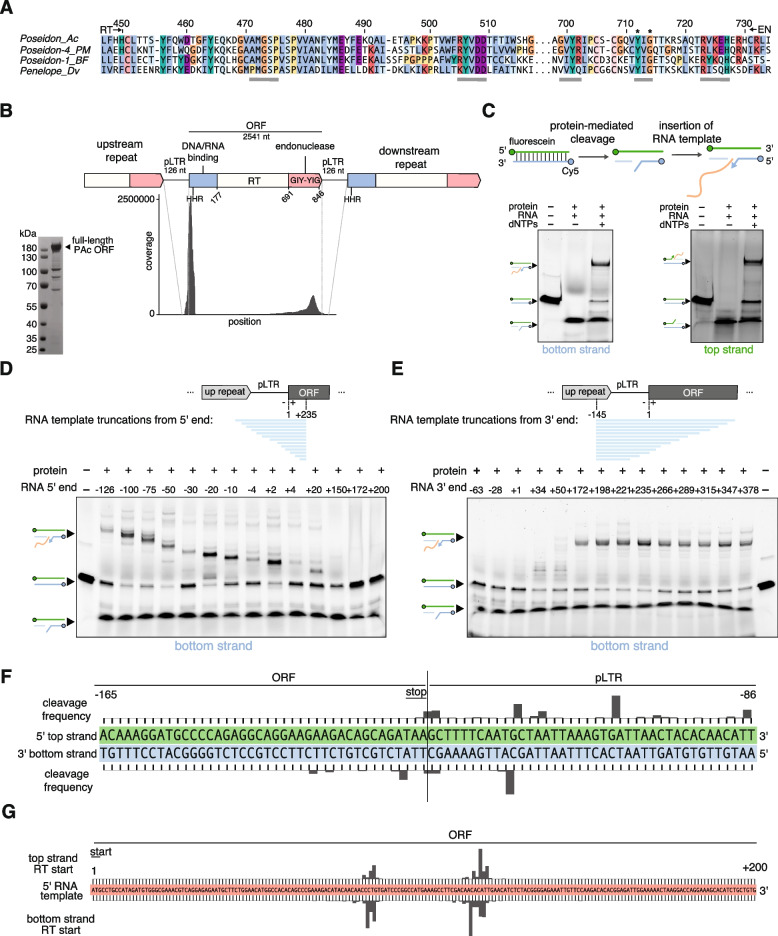
Cleavage and extension activity of the purified recombinant full-length PAc protein in vitro. **A** Amino acid sequence alignment of the core catalytic RT motifs 4 and 5 [5] (underlined) and the selected EN catalytic motifs (underlined, with mutagenized residues marked by asterisks) for four representatives from the Penelope/Poseidon PLE clade (Repbase entries from *A. carolinensis, Petromyzon marinus, Branchiostoma floridae, Drosophila virilis*); numbering on the top corresponds to PAc ORF. **B** Locus map showing repetitive structure of the PAc element. The schematic shows three example repeats with domains highlighted in blue (nucleic acid binding), white (reverse transcriptase), and red (endonuclease). pLTR, pseudo long terminal repeat; RT, reverse transcriptase; ORF, open reading frame; HHR, hammerhead ribozyme motif. Read coverage for one copy of the PAc retroelement (pLTR-ORF-pLTR from the Repbase consensus, File S1) from publicly available *A. carolinensis* RNA sequencing data is shown below the locus map. The side panel shows the degree of PAc ORF purification (Methods). **C** Overview of the in vitro assay consisting of an end-labeled dsDNA target, RNA template, and PAc protein. The schematic shows the expected effect when adding protein and RNA (cleavage), and adding protein, RNA, and dNTPs (extension). Denaturing gel shown for one RNA template for both the bottom strand (Cy5 fluorophore) and top strand (fluorescein fluorophore). The expected significance of each band as it relates to the diagram is shown by the line drawing on the left-hand side of both gels. **D** The effect of truncating the RNA template from the 5’ end on cleavage and extension activity using the in vitro assay. The 3’ end of the template is held constant at + 235 while the 5’ end is tiled from -126 to + 200 where bases are labeled relative to the start of the ORF. **E** The effect of changing the RNA template from the 3’ end on cleavage and extension activity using the in vitro assay. The 5’ end of the template is held constant at -145 while the 3’ end is tiled from -63 to + 378 where bases are labeled relative to the start of the ORF. **F** Cleavage frequency for both the top and bottom strands on a dsDNA target corresponding to the PAc ORF-pLTR junction. Bars indicate the percent of total reads mapping to a cleaved product at that location. **G** Reverse transcription initiation sites for a dsDNA target corresponding to the PAc ORF-pLTR junction and an RNA template spanning the PAc pLTR-ORF junction. Bars indicate the percent of total reads mapping to an insertion product that begins at that location

Eukaryotic retroelements are generally divided into two classes: long terminal repeat (LTR) retrotransposons and non-LTR retrotransposons. Penelope-like elements (PLEs), which are found in fish, amphibians, reptiles, insects, protists, plants, and fungi, have phylogenetically and structurally been defined as a third class of eukaryotic retroelement [1, 2]. Canonical PLEs encode a GIY-YIG endonuclease (EN) domain alongside a telomerase-like reverse transcriptase (RT) domain in a single open reading frame (ORF) [3] (Fig. 1A-B). The presence of an EN and RT domain suggests PLEs may utilize a target-primed reverse transcription (TRPT) mobilization mechanism similar to the R2 retrotransposon from *Bombyx mori* (R2Bm), where the nicked target DNA is used as a primer for reverse transcription of the transposon RNA [4]. Alternatively, since PLEs are phylogenetically distinct from both LTR and non-LTR retrotransposons, PLEs may utilize a novel mechanism of mobilization. To date, no studies have purified an active, full-length PLE protein, limiting our mechanistic understanding of these elements. Here we report the in vitro characterization of a PLE from the green anole (PAc, Poseidon from *Anolis carolinensis*), revealing mechanistic aspects not shared by other retrotransposons that explain the unusual “tail” structure observed in most of the inserted genomic copies.

PLEs are organized in tandem or partial-tandem repeats in the genomes of their host organisms; the tandem structure leads to the formation of repetitive flanking UTR sequences referred to as pseudo-LTRs (pLTRs) [1, 3, 6]. RNA sequencing from *A. carolinensis* confirmed the organization of PAc in tandem repeats at the endogenous loci (Fig. 1B). The predicted domain structure of PAc is consistent with that of canonical PLEs from the Penelope/Poseidon clade [3], consisting of a putative N-terminal nucleic acid binding domain, a central RT domain, and a C-terminal GIY-YIG EN domain. RNA sequencing reads from *A. carolinensis* (Table S1) revealed that the PAc element is expressed, with increased coverage at the pLTR-ORF junction as well as at the C-terminus of the ORF (Fig. 1B).

To detect transposition events in vitro, we developed an assay that does not introduce bias from PCR amplification (Fig. 1C). Briefly, PAc protein purified from *E. coli* is incubated with a template RNA and a dsDNA target where each strand is 5’-labelled with a different fluorophore, corresponding to the PAc ORF-pLTR junction, to reconstitute an insertion reaction. Both strands of the dsDNA target are blocked with a 3’ inverted dT to prevent non-specific extension. The end-labeled dsDNA target is then analyzed on a denaturing gel to visualize cleavage and extension activity separately for the top and bottom strands. Using this assay, we found that PAc cleaves and extends both the top and bottom strands (Fig. 1C), unlike the R2Bm and human LINE-1 retrotransposons, which have only been shown to nick and extend the bottom strand [4, 7–9].

To determine important features of the RNA template for transposition, we performed 5’ and 3’ truncations of the RNA template. Consistent with other retrotransposons, 5’ truncations reduced the length of the insertion product and showed that the full pLTR sequence is not required for insertion, with + 20 being the shortest active 5’ truncation point (numbered relative to the ORF start codon) (Fig. 1D). 3’ truncations defined the minimum RNA as requiring the first 172 nt of the ORF (Fig. 1E). We therefore hypothesize that the protein binding motif is contained within the first 172 nt of the PAc ORF. When we added additional sequences to the 3’ end of the template RNA beyond + 235 (i.e., + 266, + 289, + 315, + 347, + 378), a low intensity band was observed at the top of the gel, likely arising from priming off the 3’ end of the template; however, the dominant insertion band did not change in size with the truncations/additions, suggesting that reverse transcription initiates somewhere internal to the 3’ end of the template RNA (Fig. 1E).

**Fig. 2.**
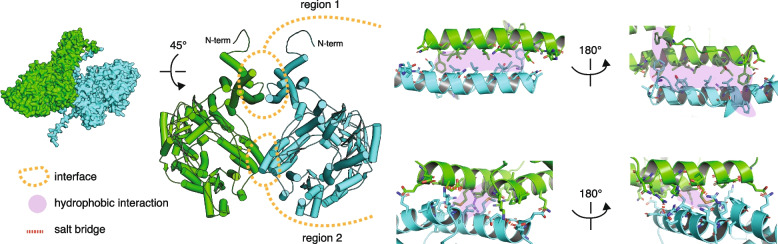
3D structure modeling of the PAc ORF. The dimeric structure was predicted using AlphaFold2 within the Colabfold framework. Three independent replicates, each with 40 cycles, were used. Each replicate consistently yielded the identical dimeric conformation, represented in green and blue (shown in surface and cartoon representations). Two key regions of interaction, outlined by orange dashed circles, stabilize the dimer. The first region features an extended helix (positions 27–173) adopting a head-to-tail homodimeric interaction, predominantly sustained by an intricate hydrophobic network. The second region consists of two helices (positions 232–250 and 795–818) from each dimeric partner, interacting head-to-tail and forming a V shape. This interaction is characterized by two symmetrical salt bridges (indicated by red dashed lines) on the periphery of the interaction patch, encasing a hydrophobic core (highlighted in purple)

We developed a strand-specific next generation sequencing (NGS) assay to more precisely probe the PAc insertion products (Fig. S1A). Analysis of cleavage sites reveals a preference for AT-rich DNA, specifically a 5’-ATT-3’ motif downstream of the ORF-pLTR junction, that represented the most frequently cleaved site on each strand (Fig. 1F). Preference for AT-rich DNA cleavage has previously been shown for a purified endonuclease domain of the Penelope element from *D. virili*s [10]; however, in contrast to the *D. virilis* endonuclease domain, PAc cleaves both the top and bottom strands. This may be due to our use of the polyprotein rather than the endonuclease domain in isolation, as the PAc polyprotein is predicted to form a dimer by AlphaFold2 (Fig. 2) [11, 12].

Analysis of RT start sites mapped from the NGS data confirms that reverse transcription preferentially initiates internal to the 3’ end of the RNA template (Fig. 1G). There does not appear to be a difference in RT start site between the top and bottom strands. Examining the relationship between the EN cut site and RT start site reveals the most common enzymatic activity as cleavage of a 5’-ATT-3’ motif followed by reverse transcription beginning in an AC-rich stem loop located 111–118 bp into the start of the ORF (Fig. S1B). Surprisingly, RT initiates internal to the 3’ end of the RNA template at a site that is not homologous to the cleaved DNA, a feature that has not been seen with other retrotransposons. Previously, the *D. virilis* Penelope EN was shown to recognize and cleave plasmids containing an equivalent Penelope fragment, and limited tiling experiments showed that the so-called “tail” sequence, an optional 30–40 bp extension found in a fraction of genomic copies, was required for EN recognition followed by cleavage, although the exact cleavage site was not defined [10]. This tail sequence clearly corresponds to the sequence between the two observed RT start sites (Fig. 1G). In Fig. S2, we document this correspondence by querying *A. carolinensis* genome assembly as well as publicly available RNA-seq reads with the template RNA sequence, and visualizing the outputs at the coverage and nucleotide level. The ends of the PAc genomic insertions (initially defining the “tail” sequence, arrows) and the ends of the corresponding RNA-seq reads fall precisely onto the RT initiation sites that are shown in Fig. 1F.

**Fig. 3:**
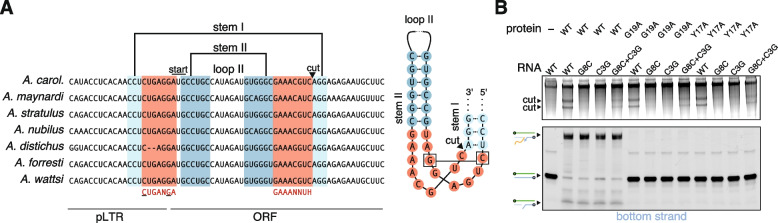
Structure and cleavage properties of the PAc hammerhead ribozyme (HHR). **A** The HHR encoded by PAc is conserved across other Anolis species. Stem I, stem II, and loop II from the canonical HHR structure along with the HHR cut site and PAc start codon are annotated. pLTR, pseudo long terminal repeat; ORF, open reading frame. Visualization of the PAc HHR secondary structure is shown next to the sequence alignment. Residues C3 and G8 in the HHR catalytic core are boxed. **B** Visualizing the role of the HHR and GIY-YIG endonuclease in RNA processing and extension in vitro. G8C and C3G are HHR mutants while G8C + C3G is a partial rescue. G19A and Y17A are key mutations in the active site of the GIY-YIG endonuclease

The PAc locus contains a hammerhead ribozyme (HHR) sequence, consistent with findings in other PLEs, containing regions corresponding to stem I, stem II, and loop II of the canonical HHR structure [13, 14]. The HHR encoded by PAc is conserved across several Anolis species (Fig. 3A). The PAc ORF begins at the start of stem II, and the HHR cut site is 26 bp downstream of the start codon. The conserved residues C3 and G8 in the HHR catalytic core occur prior to the start codon (Fig. 3A). The mutations C3G and G8C have been shown to abolish HHR cleavage activity, while the double mutant C3G/G8C partially restores ribozyme cleavage activity [15]. Consistent with this, individual G8C and C3G mutations eliminate PAc HHR activity, while the C3G/G8C double mutant partially restores cleavage activity (Fig. 3B). HHR catalytic mutants do not affect RNA insertion activity and do not affect the size of the insertion band, however, suggesting that HHR cleavage is not a necessary step for PAc-mediated transposition in vitro and that unprocessed RNA is the dominant substrate used for insertion (Fig. 3B). In the GenBank RNA-seq datasets, we failed to detect reads that begin or end at the presumptive HHR cleavage site (Fig. S2B).

Interestingly, the HHR shows no cleavage activity without the addition of protein (Fig. 3B). To confirm that RNA cleavage activity is not protein-catalyzed, we purified PAc catalytic mutants G19A and Y17A (see Fig. 1A), which have been shown to eliminate GIY-YIG endonuclease activity [16]. GIY-YIG endonuclease mutants show no DNA cleavage activity as expected, but do not impact RNA cleavage activity, suggesting that the PAc HHR is a protein-assisted ribozyme (Fig. 3B). It is possible that in vitro binding of the PAc ORF to the RT initiation site on the RNA template creates more favorable conditions for RNA folding into HHR-competent conformation (Fig. S2C), and that in vivo such conditions may arise at the later stages of the transposition cycle.

We next searched in vivo data from *A. carolinensis* to confirm our observations of AT-rich DNA cleavage followed by reverse transcription initiation in an AC-rich stem loop. We searched genome sequencing reads from the NCBI SRA database (Table S1) for motifs corresponding to a PAc insertion that initiated in the identified AC-rich stem loop and homed to an AT-rich gDNA site. For several reverse transcription initiation sites in the AC-rich stem loop, we find evidence of AT-rich homing sites (Fig. S1B).

Our results, taken together, broadly outline the following scenario of PAc mobilization. The repetitive PAc locus is transcribed and translated, likely from a promoter in its pLTR [6], leading to an mRNA consisting of multiple PAc copies alongside the PAc protein. The PAc protein binds its transposon RNA, and the PAc RNP complex then binds and nicks AT-rich gDNA, either in its own pLTR or elsewhere in the genome, and initiates reverse transcription of its template RNA within an AC-rich stem loop. Following second strand synthesis and host repair, this process eventually leads to a new PAc copy at an AT-rich gDNA sequence through additional steps that are yet to be validated experimentally. The observation that reverse transcription is initiated at a site within the transposon RNA that is not homologous to the cleaved DNA is unique to PLEs. Our system provides a controlled in vitro model to further our understanding of PLE mobilization and biology.

## Methods

### Alignment of RNA and DNA sequencing reads from *A. carolinensis*

RNA and DNA sequencing reads from *A. carolinensis* were downloaded from the NCBI Sequence Read Archive (SRA) database in FASTQ file format [17]. SRA accession numbers are included in Supplemental Table S1. Read files were aligned to the PAc locus map (Repbase ID: Poseidon_Ac; Supplemental File S1) using Bowtie 2 in “sensitive local” mode [18, 19]. Alignment files in .sam format were concatenated and parsed to isolate CIGAR strings. A match character (“M”) was used to sum coverage across the PAc reference map for all CIGAR strings.

Alignment of representative Repbase sequences from the Penelope/Poseidon clade in Fig. 1A was generated with MAFFT v.7 and visualized with Jalview v. 2.11.3.2 using the Clustal color scheme [20, 21].

### Molecular cloning of plasmids

PLE sequences were downloaded from the Repbase database (PAc Repbase ID: Poseidon_Ac) and cloned by Genscript [22, 23]. Point mutations (e.g., G19A, Y17A) were generated by KLD cloning (New England Biolabs). Several iterations of N and C-terminal solubility and affinity tags were cloned by Gibson Assembly (New England Biolabs). All plasmid sequences were verified via Tn5 tagmentation and next generation sequencing (Illumina) [24].

### Recombinant protein expression in *E. coli*

An inducible bacterial expression plasmid coding for the PAc ORF with an N-terminal 14 × His-MBP tag and C-terminal TwinStrep tag was transformed into Rosetta(DE3) competent cells (Millipore Sigma). A single colony was picked and placed in 33 mL of starter for overnight incubation. 5 mL of starter was used to inoculate 1 L of media. Cultures were grown at 37 °C in Terrific Broth (Thermo Fisher Scientific) until OD_280 nm_ = 0.8. Cultures were then cooled to 18 °C, and protein expression was induced with the addition of Isopropyl β-D-1-thiogalactopyranoside (IPTG) (Gold Biotechnology) to a final concentration of 250 μM followed by 18 h of growth at 18 °C. Cells were harvested by centrifugation at 5000 g for 10 min, and pellets were resuspended in 50 mM Tris–HCl pH 7.5, 1 M NaCl, 10% glycerol, 5 mM beta-mercaptoethanol (BME), and cOmplete ULTRA EDTA-free protease inhibitor (Roche). Cells were lysed using an LM20 microfluidizer (Microfluidics), and lysate was cleared by centrifugation at 18,000 RPM for 20 min. Cleared lysate was bound to Strep-Tactin Superflow Plus (Qiagen) resin at 4 °C for 60 min with rotation. The resin was washed several times at 4 °C, and then washed one final time with elution buffer (20 mM HEPES–KOH pH 7.9, 500 mM KCl, 10% glycerol, and 1 mM tris carboxyl ethyl phosphene (TCEP)). Resin was eluted 10 times with 1 mL elution buffer supplemented with 5 mM d-Desthiobiotin (Millipore Sigma). Elutions were run on an SDS-PAGE gel followed by Coomassie blue staining, and elutions containing high concentrations of proteins were pooled, aliquoted in 100 μL, snap frozen in liquid nitrogen, and stored at -80 °C. From 12 L, we obtained ~ 2.5 mg purified Pac protein.

### In vitro cleavage and extension assays

In vitro reactions were performed in 20 mM HEPES–KOH pH 7.9, 400 mM KAc, 100 mM KCl, 5% glycerol, 5 mM MgAc, 0.2 mM TCEP, and 1 mM d-Desthiobiotin. Reactions contained 500 nM PAc protein, 500 nM RNA template, and 10 nM labeled dsDNA target. RNA templates for in vitro transcription (IVT) were produced by PCR with a T7 promoter added to the forward primer. For IVT, PCR reactions were diluted 1/10 in the reaction mixture containing 4 mM each NTP, 20 mM MgCl_2_, 40 mM Tris–HCl pH 8.0, 10 mM DTT, 1 mM spermidine, 85 μg/mL of homemade T7 RNA polymerase, and incubated at 37 °C for 90 min. The pyrophosphate precipitate was pelleted and removed, and reactions were treated with 1/100 vol of RNase-free DNase I (NEB) at 37 °C for 15 min. The 361-nt RNA template used in most assays contained the 126-nt UTR plus the first 235 nt of the PAc ORF from the Poseidon_Ac Repbase consensus (gcuuuucaaugcuaauuaaagugauuaacuacacaacauucacacugaccucucucacccuagacuuuccacagauauauauuaaccucuuugcuuaguuuucuccauaccucacaaccucugaggAUGccugccauagaugugggcgaaacgucaggagagaaugcuucuggaacauggccacacagcccgaaagacauacaacaacccugugaucccggccaugaaagccuucgacaacacauugaacaucucuacggggagaaauuguuccaagacacacggagauuggaaaaacuaaggaccaggaaagcacaucugcugugcucccugaccuuccuucuacgcugcagagacacag) (full consensus in file S1). dsDNA target was prepared by annealing a labeled top strand (/56-FAM/acaaaggatgccccagaggcaggaagaagacagcagataagcttttcaatgctaattaaagtgattaactacacaacatt/3InvdT/) and a labeled bottom strand (/5Cy5/aatgttgtgtagttaatcactttaattagcattgaaaagcttatctgctgtcttcttcctgcctctggggcatcctttgt/3InvdT/). Reactions were incubated at 32 °C for 90 min followed by mixing with equal volumes of 2X TBE-Urea sample buffer (90 mM Tris base, 90 mM boric acid, 2 mM EDTA, 12% Ficoll Type 400, 7 M urea, and 0.02% bromophenol blue). The chosen temperature (32 °C) corresponds to the average temperature experienced by anole lizards in their natural environments [25]. Reactions were then boiled at 95 °C for 3 min, followed by running on a precast 10% TBE-Urea gel (Invitrogen) at 400 V for 12 min. Fluorescent signals in all gels were visualized using a ChemiDoc (BioRad).

### In vitro NGS assay

In vitro reactions were performed as described above, except the dsDNA target was biotinylated on either the top strand or bottom strand rather than end-labeled with a fluorophore. Biotinylated DNA primers were ordered (Integrated DNA Technologies) and used in a PCR with Q5 Hot Start High-Fidelity polymerase (New England Biolabs) to create a dsDNA target. Reactions were stopped with the addition of 5 μL RNase A (New England Biolabs) followed by incubation at 37 °C for 30 min. A phenol chloroform extraction was then performed with UltraPure Phenol:Chloroform:Isoamyl Alcohol (Thermo Fisher Scientific) followed by ethanol precipitation and resuspension in 2X TBE-Urea sample buffer (90 mM Tris base, 90 mM boric acid, 2 mM EDTA, 12% Ficoll Type 400, 7 M urea, and 0.02% bromophenol blue). Reactions were gel extracted from a 10% TBE-Urea gel and passed through a 1 mL syringe to shear gel fragments. Sheared gel fragments were incubated overnight at 4 °C in 0.3 M sodium acetate pH 5.5 and 10 mM EDTA to allow diffusion of the DNA out of the gel. Another ethanol precipitation was performed prior to ligation. A 5’ adenylated and 3’ capped adapter (/5rApp/CTGTCTCTTATACACATCTCCGAGCCCACGAGAC/3SpC3/) was ligated to purified DNA with a thermostable 5’ App DNA/RNA ligase (New England Biolabs) at 65 °C for 16 h. Following proteinase K (New England Biolabs) treatment, ligated DNA was immobilized on Dynabeads M-270 Streptavidin (Thermo Fisher Scientific) by incubation at room temperature with agitation for 30 min in binding/wash buffer (5 mM Tris–HCl pH 7.5, 0.5 mM EDTA, and 1 M NaCl). The beads were washed several times with binding/wash buffer to remove excess adapter. Beads were input directly into a PCR with KAPA HiFi HotStart ReadyMix (Roche) for 30 cycles. NGS libraries were gel extracted and quantified using Qubit Fluorometric Quantification (Thermo Fisher Scientific). Libraries were sequenced on a MiSeq (Illumina) with 100 cycles read 1, 8 cycles index 1, 8 cycles index 2, and 100 cycles read 2 supplemented with 10% PhiX Control v3 (Illumina) for diversity.

### Processing NGS data

NGS data was first trimmed for low quality reads (Q-score < 30), followed by removing reads that did not contain the expected adapter sequence at the start of the read. Trimmed FASTQ files were aligned to the dsDNA target reference map to identify the cleavage site using Bowtie 2 in “local” mode [18, 19]. Sequences in the read beyond the dsDNA cleavage site were assumed to be RNA-templated insertions, and these sequences were similarly mapped to an RNA template reference map to determine the site of reverse transcription initiation.

### In vitro HHR assay

In vitro HHR reactions were performed in 20 mM HEPES–KOH pH 7.9, 400 mM KAc, 100 mM KCl, 5% glycerol, 5 mM MgAc, 0.2 mM TCEP, and 1 mM d-Desthiobiotin. Reactions contained 500 nM PAc protein and 500 nM RNA (-126/ + 235). Reactions were incubated at 32 °C for 2 h followed by mixing with equal volumes of 2X TBE-Urea sample buffer (90 mM Tris base, 90 mM boric acid, 2 mM EDTA, 12% Ficoll Type 400, 7 M urea, and 0.02% bromophenol blue). Reactions were then boiled at 95 °C for 3 min, followed by running on a precast 10% TBE-Urea gel (Invitrogen) at 400 V for 12 min. Previously, we observed self-cleavage in PLE HHR at Mg^2+^ concentrations varying from 3 to 25 mM [26]. 

### Supplementary Information

Supplementary Material 1: Fig. S1. Details of the NGS assay and cleavage/insertion site localization on PAc RNA. (A) Schematic of the workflow for an NGS assay used for strand-specific sequencing of reaction products. Biotinylated dsDNA is incubated with PAc protein, template RNA and dNTPs as described in Methods, extracted from a denaturing gel, immobilized on streptavidin beads, adapter ligated, and amplified prior to sequencing. (B) Predicted RNA secondary structure for the conserved RNA sequence at the start of the PAc ORF. Shapes indicate the highest frequency reverse transcription initiation sites as predicted from the NGS data shown in Fig. 1F**,** while pink circles indicate all reverse transcription initiation sites (i.e. “tail” boundaries). WebLogo [27] plots are shown for AT-rich homing sites for the reverse transcription initiation sites as calculated from gDNA sequencing data from *A. carolinensis *(Table S1). Shapes correspond to the start sites shown on the RNA secondary structure prediction by RNAfold [28] .

Supplementary Material 2: Fig. S2. Visualization of gDNA and RNA sequences homologous to the N-terminal part of PAc ORF. GenBank databases were queried with nt -100 to +200 relative to the PAc AUG codon, so that the numbers shown on the top represent the consensus PAc numbering used throughout the text plus 100, i.e. the approx. 34-nt “tail” sequence roughly spans nt 85-119 of the consensus (vertical arrows). Screenshots display sequence alignments for a 150-bp window in the NCBI MSA Viewer 1.25.0; plots on the top show query coverage and the number of reads at peak coverage. (A) NCBI megablast search of the *A. carolinensis* WGS assembly AAWZ; (B) Example of a megablast search of the *A. carolinensis* 150-nt RNA-seq SRA reads [29] from Table S1 (accession SRR14288908) with ‘max target sequences’ set at 5000. The rightmost part of the alignment shows the reads extending from the “tail” region into the body of the element, indicating ongoing transcription of full-length copies not visible in the plot in Fig. 1A due to much higher coverage in the pLTR region. Position 30 (#130 on the figure) corresponds to the expected HHR cleavage site (asterisk), however there are no reads beginning or ending at this site, and the coverage plots on the top indicate no discontinuities in this region. Other SRA accessions display similar patterns. (C)  Alternative PAc RNA structure predictions using the deep-learning-based MXfold2 server [30]. The top RNA and the RT start sites are the same as in Fig. S1B, beginning with the presumed HHR cleavage site. The bottom RNA includes the uncleaved HHR motif as shown in Fig. 3 (PAc nt -9 to +44) with folded stem-loop II, and outlines the hypothetical interaction area with PAc RT moiety (cloud-like) in a large loop near the first RT start site, which needs to undergo unfolding of the conserved HHR catalytic core (nt -6 to 1) for reverse transcription to occur through it. Gray lines indicate base-pairing that would be required to form HHR stem I. The expected cleavage site is indicated between C29-A30.

Supplementary Material 3: Table S1. SRA accessions used in preparing Fig. S1B (DNA), Fig. 1B and Fig. S2 (RNA).

Supplementary Material 4: File S1. Consensus sequence of the Poseidon_Ac entry from Repbase [18] (in Genbank format). The database requires subscription since 2019, however this entry was assembled from Sanger reads and deposited by I.A. as part of ref [19] under initial assumption that it would be freely available to researchers.

## Data Availability

All data generated or analysed during this study are included in this published article and its supplementary information files. Materials can be obtained from Addgene.

## Abbreviations

EN: Endonuclease
gDNA: Genomic DNA
HHR: Hammerhead ribozyme
NGS: Next generation sequencing
ORF: Open reading frame
PAc: Poseidon *A. carolinensis*
PLE: Penelope-like elements
pLTR: Pseudo (or Penelope) long terminal repeat
RNP: Ribonucleoprotein
RT: Reverse transcription
SRA: Short read archive
TPRT: Target-primed reverse transcription
