# Supplementary figures and images for: Internal initiation of reverse transcription in a Penelope-like retrotransposon
Source: Mob DNA. 2024 Jun 11;15:12. doi: 10.1186/s13100-024-00322-z (PMC11167929; doi:10.1186/s13100-024-00322-z)

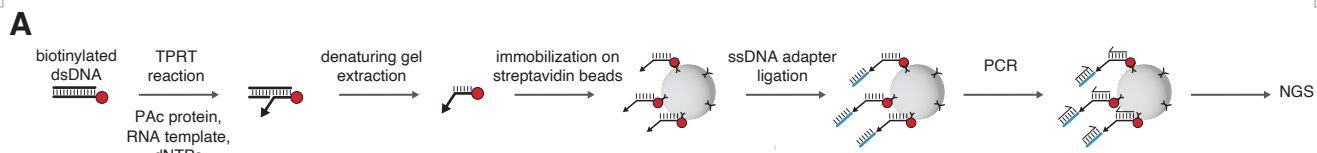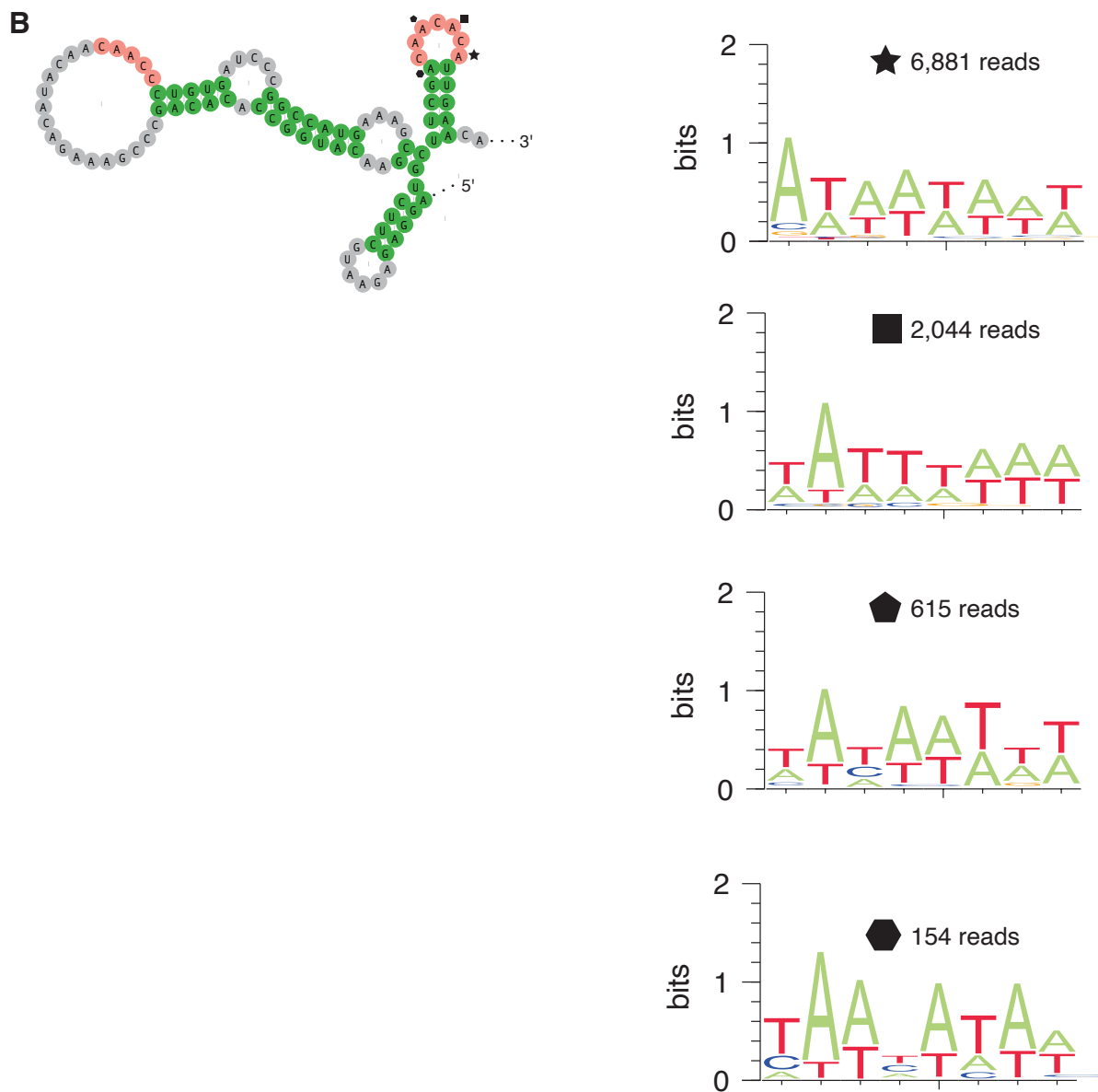

**Supplementary Figure 1**

Supplement: Supplementary file 1 — Supplementary Material 1: Fig. S1. Details of the NGS assay and cleavage/insertion site localization on PAc RNA. (A) Schematic of the workflow for an NGS assay used for strand-specific sequencing of reaction products. Biotinylated dsDNA is incubated with PAc protein, template RNA and dNTPs as described in Methods, extracted from a denaturing gel, immobilized on streptavidin beads, adapter ligated, and amplified prior to sequencing. (B) Predicted RNA secondary structure for the conserved RNA sequence at the start of the PAc ORF. Shapes indicate the highest frequency reverse transcription initiation sites as predicted from the NGS data shown in Fig. 1F, while pink circles indicate all reverse transcription initiation sites (i.e. “tail” boundaries). WebLogo [27] plots are shown for AT-rich homing sites for the reverse transcription initiation sites as calculated from gDNA sequencing data from A. carolinensis (Table S1). Shapes correspond to the start sites shown on the RNA secondary structure prediction by RNAfold [28] . [file 13100_2024_322_MOESM1_ESM.pdf]

A

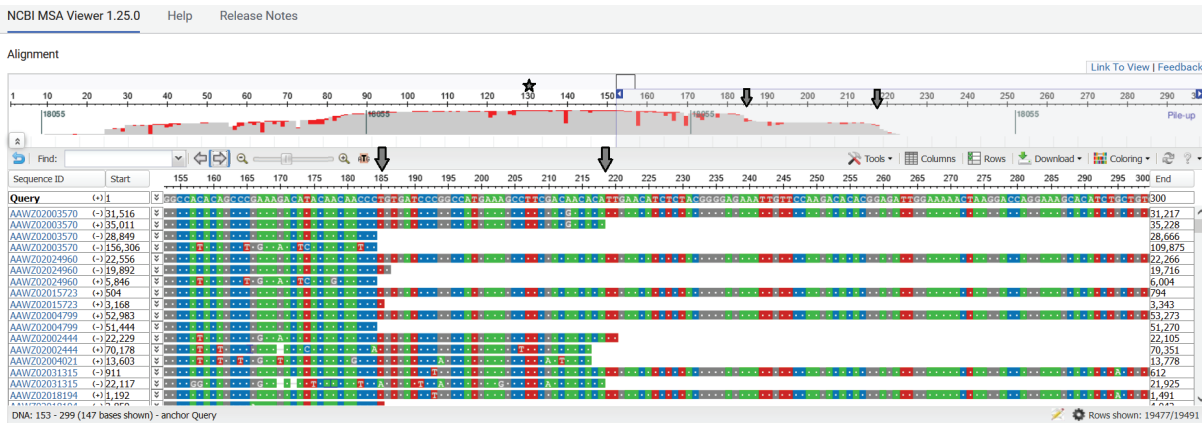

B

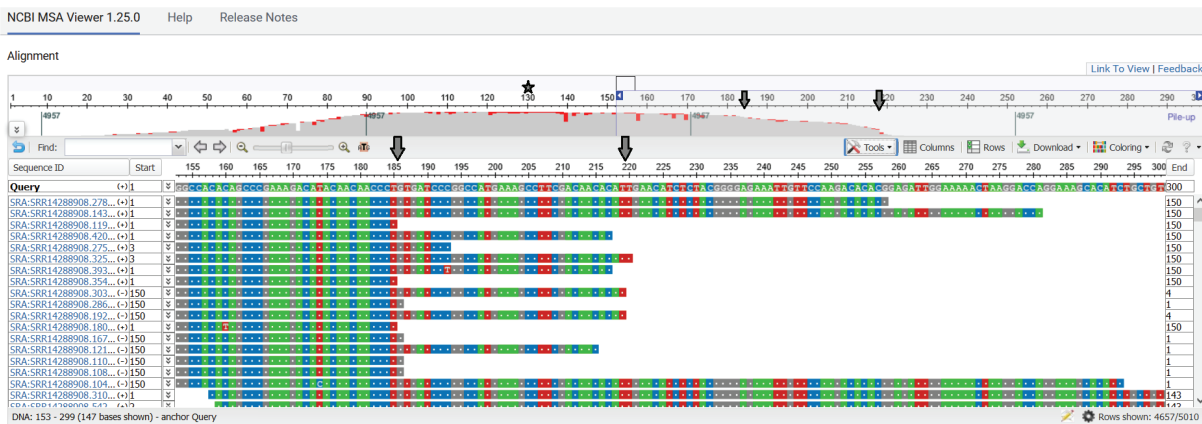

C

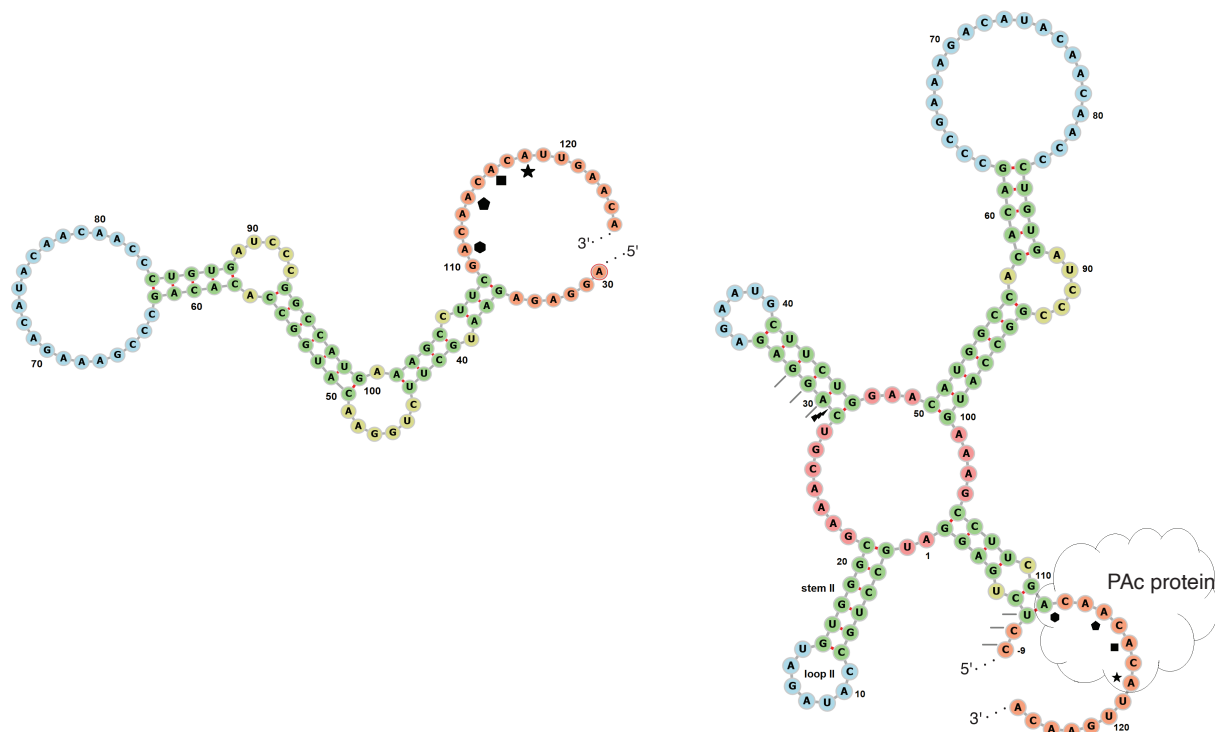

# Supplementary Figure 2

Supplement: Supplementary file 2 — Supplementary Material 2: Fig. S2. Visualization of gDNA and RNA sequences homologous to the N-terminal part of PAc ORF. GenBank databases were queried with nt -100 to +200 relative to the PAc AUG codon, so that the numbers shown on the top represent the consensus PAc numbering used throughout the text plus 100, i.e. the approx. 34-nt “tail” sequence roughly spans nt 85-119 of the consensus (vertical arrows). Screenshots display sequence alignments for a 150-bp window in the NCBI MSA Viewer 1.25.0; plots on the top show query coverage and the number of reads at peak coverage. (A) NCBI megablast search of the A. carolinensis WGS assembly AAWZ; (B) Example of a megablast search of the A. carolinensis 150-nt RNA-seq SRA reads [29] from Table S1 (accession SRR14288908) with ‘max target sequences’ set at 5000. The rightmost part of the alignment shows the reads extending from the “tail” region into the body of the element, indicating ongoing transcription of full-length copies not visible in the plot in Fig. 1A due to much higher coverage in the pLTR region. Position 30 (#130 on the figure) corresponds to the expected HHR cleavage site (asterisk), however there are no reads beginning or ending at this site, and the coverage plots on the top indicate no discontinuities in this region. Other SRA accessions display similar patterns. (C) Alternative PAc RNA structure predictions using the deep-learning-based MXfold2 server [30]. The top RNA and the RT start sites are the same as in Fig. S1B, beginning with the presumed HHR cleavage site. The bottom RNA includes the uncleaved HHR motif as shown in Fig. 3 (PAc nt -9 to +44) with folded stem-loop II, and outlines the hypothetical interaction area with PAc RT moiety (cloud-like) in a large loop near the first RT start site, which needs to undergo unfolding of the conserved HHR catalytic core (nt -6 to 1) for reverse transcription to occur through it. Gray lines indicate base-pairing that would be required to form HHR stem I. The [file 13100_2024_322_MOESM2_ESM.pdf]
